# Incongruent Nuclear and Mitochondrial Genetic Structure of New World Screwworm Fly Populations Due to Positive Selection of Mutations Associated with Dimethyl- and Diethyl-Organophosphates Resistance

**DOI:** 10.1371/journal.pone.0128441

**Published:** 2015-06-01

**Authors:** Luana Walravens Bergamo, Pablo Fresia, Ana Maria L. Azeredo-Espin

**Affiliations:** 1 Center for Molecular Biology and Genetic Engineering (CBMEG), Campinas State University (UNICAMP), Campinas, SP, Brazil; 2 Department of Genetics, Evolution and Bioagents (DGEB), Institute of Biology (IB), Campinas State University (UNICAMP), Campinas, SP, Brazil; Australian National University, AUSTRALIA

## Abstract

Livestock production is an important economic activity in Brazil, which has been suffering significant losses due to the impact of parasites. The New World screwworm (NWS) fly, *Cochliomyia hominivorax*, is an ectoparasite and one of the most important myiasis-causing flies endemic to the Americas. The geographic distribution of NWS has been reduced after the implementation of the Sterile Insect Technique (SIT), being eradicated in North America and part of Central America. In South America, *C*. *hominivorax* is controlled by chemical insecticides, although indiscriminate use can cause selection of resistant individuals. Previous studies have associated the Gly137Asp and Trp251Leu mutations in the active site of carboxylesterase E3 to resistance of diethyl and dimethyl-organophosphates insecticides, respectively. Here, we have sequenced a fragment of the carboxylesterase E3 gene (*ChαE7*), comprising part of intron iII, exon eIII, intron iIII and part of exon eIV, and three mitochondrial gene sequences (CR, COI and COII), of NWS flies from 21 locations in South America. These markers were used for population structure analyses and the *ChαE7* gene was also investigated to gain insight into the selective pressures that have shaped its evolution. Analysis of molecular variance (AMOVA) and pairwise FST analysis indicated an increased genetic structure between locations in the *ChαE7* compared to the concatenated mitochondrial genes. Discriminant analysis of principal components (DAPC) and spatial analysis of molecular variance (SAMOVA) indicated different degrees of genetic structure for all markers, in agreement with the AMOVA results, but with low correlation to geographic data. The NWS fly is considered a panmitic species based on mitochondrial data, while it is structured into three groups considering the *ChαE7* gene. A negative association between the two mutations related to organophosphate resistance and Fay & Wu’s H significant negative values for the exons, suggest that these mutations evolved under positive selection.

## Introduction

Livestock production is an important economic activity in Brazil that is strongly affected by parasites. The New World screwworm (NWS), *Cochliomyia hominivorax* (Diptera: Challiphoridae) [1], is a myiasis-causing fly endemic from the Americas that is one of the main ectoparasites [2,3]. Larvae of the NWS fly feed on living tissues from natural cavities and wounds of warm-blooded vertebrates, mostly of domestic animals.

The current distribution of the NWS fly is neotropical, including Cuba, the Dominican Republic, Haiti, Jamaica and all South American countries [4]. Originally, the NWS fly was distributed from southern United States of America to Argentina and Uruguay [2–4] but the implementation of an area-wide integrated management approach (AW-IPM) based on the Sterile Insect Technique (SIT) reduced its distribution [4–6]. Because of the success of this approach, the implementation of a NWS fly control program in its current distribution is under discussion [7]. There are many crucial biological questions involving the management of insect pest populations that should be investigated in addition to political decisions and interests in order to achieve the objectives. These include understanding population distribution, detecting geographic barriers or environmental discontinuities limiting gene flow and determining population sizes in target areas [7,8].

A fundamental factor for effectively applying the SIT program against NWS fly is the low density of target populations [9]. The first step of such a program relies on the use of insecticides to decrease population sizes. However, it will not be effective if populations have a high insecticide resistance level. In Brazil, like in other South American and Caribbean countries, ectoparasite control is performed exclusively by the use of chemical insecticides [10]. However, the indiscriminate use of these compounds has resulted in an increased frequency of resistant individuals and a consequent decreased efficacy [11,12].

The molecular basis of resistance mechanisms involve alterations in target sensitivity and/or metabolic detoxification of the chemical compound before it hits the target [13]. An example of this is related to acetylcholinesterase (AChE), which has decreased sensitivity to carbamates and organophosphates insecticides, leading to resistance in many insect species [14–17]. Previous studies have associated changes in ‘ali-esterase’ activity of carboxylesterase E3, from carboxylesterase to organophosphate hydrolase, with two types of organophosphate insecticide resistance in *Chrysomya putoria*, *Musca domestica* and *Lucilia cuprina* [18]. Both resistance types, named ‘diazinon’ and ‘malathion’, confer broad spectrum resistance to OPs. However, ‘diazinon’ shows high resistance levels to diethyl OPs and low to dimethyl OPs, while ‘malathion’ shows high resistance levels to dimethyl OPs, specially to malathion, and low to diethyl OPs. ‘Ali-esterase’ and OP hydrolase activities are reduced and increased in both resistance types, respectively. Malathion carboxylesterase (MCE) activity, which is the hydrolysis of the carboxylester groups of malathion, is high in ‘malathion’ and low in ‘diazinon’ resistance type [18–21]. The increased resistance to diethyl-organophosphates in ‘diazinon’ resistance type is conferred by the Gly137Asp mutation in the oxyanion hole of the carboxylesterase E3 [22], while resistance to dimethyl-organophosphates in ‘malathion’ resistance type is conferred by the Trp251Leu mutation in the acyl pocket of the enzyme target site [21], which is also associated to pyrethroids resistance [23]. The two mutations could not be present in the same allele by recombination, once the Gly137Asp mutation abolishes the enzymes native MCE activity, which is fundamentally necessary for malathion resistance [21]. However, double resistance can be found due to duplications of esterase gene, with each copy of the gene carrying each resistance type [24].

The first molecular structure of an insect carboxylesterase E3 protein was recently solved for *L*. *cuprina* (*LcαE7*) and elucidates the function of this enzyme, both in its native (associated with lipids metabolism) and phosphorylated (associated to OP resistance) forms, enabling a better understanding of the effects of both mutations on the wild-type function of the enzyme [25].

The two mutations (Gly137Asp and Trp251Leu) were identified in the NWS fly and it is hypothesized that these confer resistance through a similar mechanism based on homology with mutations in other species for which resistance has been proven through bioassays [18,22,26–28]. Because of the absence of a susceptible lineage, bioassays are difficult to conduct in NWS fly and the detection of positive selection signals in field populations represents an alternative approach to indirectly demonstrate the association of both mutations with insecticide resistance [29,30].

Another important factor to successfully implement a control program based on SIT is the adequate delimitation of the geographic areas to be targeted [31]. Many population genetic studies, based on several molecular markers, were conducted with this aim in the NWS fly, which resulted in different population structure scenarios and interpretations [32–39]. At least four genetic regional groups were described for the NWS fly throughout its current geographical range, including Cuba (CG), the Dominican Republic (DRG), and North and South of the Amazon Region (NAG and SAG, respectively), with island populations being derived from mainland ones [38]. In the mainland, a split between populations from North/Central America and South America, preceded by an expansion process initiated in North America, occurred after the Last Glacial Maximum. A subsequent split between the Pleistocene and Holocene epochs resulted in the NAG and SAG regions in South America [39]. Within these two areas, the NWS fly did not share mitochondrial haplotypes [38] and the genetic structure was associated with a barrier in the north of the Amazon basin, with NWS fly populations from the Amazon only sharing haplotypes with those in the SAG but not in the NAG region [40]. Despite the wide area sampled, a low population differentiation was observed without a geographic correlation in the SAG group [38], probably due to the population expansion process [39].

In this study, we characterized a fragment of carboxylesterase E3 gene (*ChαE7*) and investigated the geographic distribution of its genetic variability in NWS fly populations from SAG group locations. We also carried out comparative analyses of *ChαE7* variability by also investigating the same parameters of mitochondrial genes. Neutrality tests based on frequency spectrum and linkage disequilibrium tests were also used to detect positive selection signals in the mutations associated with insecticide resistance.

## Materials and Methods

### Ethics Statement

Samples were obtained in private farms and no specific permissions were required for the present studies. Additionally, we confirm that the field studies did not involve endangered or protected species.

### Carboxylesterase E3 (*ChαE7*) gene characterization

**Fig 1:**
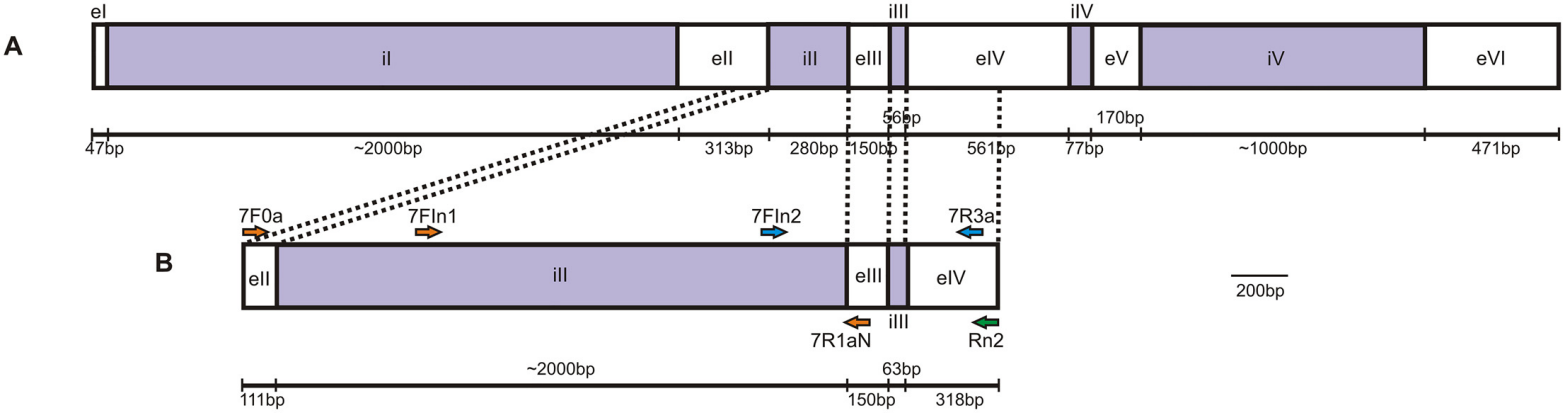
Carboxylesterase E3 *ChαE7* schematic view. Comparison of exon and intron lengths and position between (A) *L*. *cuprina* and (B) *C*. *hominivorax*. Exons and introns were named according to *L*. *cuprina* (e = exon; I = intron) [27]. The position of primers used for gene region characterization (7F0a, RN2, 7FIn1 and 7R1aN) and for posterior populational analyses (7FIn2, RN2 and 7R3a) are indicated by blue and red arrows, respectively. RN2 is indicated by a green arrow (used for both characterization and population analyses).

The total genomic DNA of five NWS fly individuals from Brazil (2) and Uruguay (3) was extracted by the phenol:chloroform method with modifications [36]. Polymerase chain reaction (PCR) amplification of a *ChαE7* fragment comprising intron iII, exon eIII, intron iIII and part of exon eIV (Fig 1) was carried out initially using the specific primers 7F0a (5´-GGTATACCATACGCCCAAC -3´) and RN2 (5´-AACAGTAATCCCTCGTACG-3´). Both primers were designed based on NWS fly cDNA [41] using Primer 3 software [42]. PCR amplifications were conducted in 15μL containing 10X PCR buffer, 200 μM of each dNTP, 1 unit of bovine serum albumin (BSA, New England Biolabs, Ipswich, MA, USA), 2.5 μM MgCl_2_, 1 μM of each primer, 1 unit of *Taq* DNA polymerase (Fermentas International Inc., Canada) and ~15–20 ng of DNA. Amplifications were performed with an initial denaturing step of 3 min at 96°C, followed by 35 cycles of 1 minute at 95°C, 1 minute at 62°C and 2 minutes at 72°C, and ending with a final elongation step of 10 minutes at 72°C. The reaction products were sequenced and used for nested PCR with new primers (7FIn1: 5´-ATTGTGTCTCCCTGCAAGTG-3´ and 7R1aN: 5´-CGTTTAGTTTCTGGAGCC-3´) based on the obtained sequences. The nested PCRs were conducted as above but with a reduced MgCl_2_ concentration (1.5 μM) and an annealing temperature of 52°C. PCR products were purified with the Illustra GFX PCR DNA and Gel Band purification Kit according to manufacturer’s instructions (GE Healthcare, Little Chalfont, UK) and cloned into the pGEM-T vector (Promega Co., Madison, WI, USA). Clones were sequenced with forward and reverse M13 primers using the Big Dye Terminator Cycle Sequencing ABI Kit 3.1 in an automatic sequencer ABI 3700 (Applied Biosystems, Inc.; Foster City, CA, USA). Chromatograms were inspected with FinchTV 1.4.0 software (Geospiza Inc., Seattle, WA, USA) taking into account Phred values [43,44]. Contiguous, overlapping bidirectional sequences (contigs) of each individual were obtained using CAP3 software [45], aligned with the algorithms of ClustalX2 [46] and Muscle [47], as implemented in Mega 5.2 software [48], and manually edited. Sequence length and nucleotide composition (A%, T%, C%, G%, A+T%, C+G%, AT-skew = [A-T]/[A+T] and GC-skew = [G-C]/[G+C]) of forward strands were compared with *Lucilia cuprina* (GenBank accession numbers: U56636 and AY691508), *Drosophila melanogaster* (GenBank accession number: NM079537) and *Musca domestica* (GenBank accession number: AF133341).

### Population samples and sequencing

Stored genomic DNA (-80°C) from 201 NWS fly larvae collected in 21 sampling locations from South America, previously analyzed in [36] and [38], were used in this study (Table 1).

**Table 1:** NWS fly sampling locations.

Country	ID	Location	State/District	Latitude	Longitude	N
Brazil	BTO	Touros	Rio Grande do Norte	05° 17'S	35° 33'W	9
	BGN	Goianira	Goiás	16° 32'S	49° 22'W	10
	BGO	Goiânia	Goiás	16° 43'S	49° 15'W	10
	BCA	Caiapônia	Goiás	16° 57'S	51° 48'W	10
	BCR	Costa Rica City	Mato Grosso do Sul	18° 32'S	53° 07'W	10
	BAQ	Aquidauana	Mato Grosso do Sul	19° 35'S	56° 05'W	10
	BSS	São Seb. Paraíso	Minas Gerais	20° 55'S	46° 59'W	10
	BES	Estiva	Minas Gerais	22° 27'S	46° 01'W	9
	BCI	Carambeí	Paraná	24° 55'S	50° 05'W	12
	BFV	Fagundes Varela	Rio Grande do Sul	28° 52'S	51° 41'W	9
	BSA	Sto. Ant. Missões	Rio Grande do Sul	29° 04'S	56° 19'W	10
	BPM	Pinheiro Machado	Rio Grande do Sul	31° 34'S	53° 23'W	11
Paraguay	PYB	Ybytymi	Paraguarí	25° 46'S	56° 41'W	10
Uruguay	UPM	Paso Muñoz	Salto	31° 27'S	56° 23'W	10
	UST	San Antonio	Salto	31° 24'S	57° 58'W	10
	UDA	Daymán	Paysandú	31° 33'S	57° 57'W	7
	UBM	Bañado de Medina	Cerro Largo	32° 23'S	54° 21'W	11
	UCC	Cerro Colorado	Florida	33° 52'S	55° 33'W	10
	UCO	Colonia	Colonia	34° 28'S	57° 51'W	10
	UJS	Juaquín Suarez	Canelones	34° 44'S	56° 02'W	9
Argentina	APL	Lezama	Buenos Aires	35° 52'S	57° 53'W	4

ID = site identification code; N = number of sampled individuals.

Fragments of three mitochondrial genes, the *cytochrome c oxidase* subunit I (COI) and subunit II (COII) and the B domain of the control region (CR), were amplified and sequenced using the same protocols and conditions for 54 flies that were not previously analyzed by [38]. Part of the *ChαE7* fragment previously characterized, including 157 bp from intron iII, the exon eIII (150 bp), the intron iIII (63 bp) and 282 bp from exon eIV, was amplified from all the individuals using the forward primer 7FIn2 (5´-ACCATCGGTGAGTTGAGAG-3´) and reverse primers RN2 (5´-AACAGTAATCCCTCGTACG-3´) or 7R3a (5´-ATCCTTATCATTATTTTCACCC -3´) (Fig 1), leading to a predicted fragment size of 652 bp. The forward primer (7Fln2) was designed based on sequences previously obtained in this study, with Primer 3 software [42]. The reverse primer 7R3a was designed as described previously [28]. PCR amplifications were performed in a final volume of 25μL containing 10X PCR buffer, 100 μM of each dNTP, 1 unit of bovine serum albumin (BSA, New England Biolabs, Ipswich, MA, USA), 2.5 mM MgCl_2_, 0.2 μM of each primer, 1 unit of *Taq* DNA polymerase (Fermentas International Inc., Canada) and ~15–20 ng of DNA. An initial denaturing step of 3 minutes was performed at 96°C, followed by 35 cycles of 1 minute at 95°C, 1 minute at 56°C and 2 minutes at 72°C, and a final elongation step of 10 minutes at 72°C. The PCR products were purified and sequenced as before. Sequences were trimmed, evaluated for quality and assembled in contigs using the Geneious 6.0.6 software (Biomatters Ltd.; Auckland, NZ). When heterozygotes with alleles containing insertions/deletions (indels) of different lengths within intron iII were detected, PCR amplifications were repeated and the products were purified and cloned into the pGEM-T vector (Promega Corp). At least 6 clones from each individual were sequenced, in order to obtain sequences from both alleles.

### Sequences analyses

Sequences from all the 201 individuals were aligned with the algorithms of ClustalX2 [46] and Muscle [47], manually edited in Mega 5.2 [48] and inspected for quality with the Gblock Server 0.91b [49]. Mitochondrial sequences were aligned considering each fragment separately (CR, COI and COII) and subsequently concatenated for analyses. COI, COII and *ChαE7* exons (eIII and eIV) were checked for open reading frames using Mega 5.2 [48]. For the CR fragment and *ChαE7* intron iII sequences, each indel was considered as a single mutation and all indels were recoded as single positions in the final alignment. Individual sequences were collapsed into unambiguous haplotypes using FaBox [50], and these were compared and named according to sequences reported previously in the case of the mitochondrial data [38–40].

### Population analyses

General diversity indices, such as number of haplotypes, haplotype diversity or expected heterozygosity (Ĥ) and nucleotide diversity (π) [51], were calculated for each dataset (mitochondrial CR, COI, COII and nuclear *ChαE7*) independently using the DnaSP 5.0 [52] and Arlequin 3.5 [53] softwares.

The genetic structure of NWS fly populations was first investigated using the Analysis of Molecular Variance (AMOVA) and pairwise F_ST_. This was performed using the Arlequin 3.5 software [53] separately for each dataset and considered each sampled location as a population and all as belonging to a unique population. The statistical significance values were accessed using 10,000 permutations. We also carried out a discriminant analysis of principal components (DAPC) [54] implemented in adegenet 1.3–8 from the R package [55]. This is a multivariate analysis without an *a priori* population model that identifies clusters (K) of individuals independent from geographic origin. The analysis was performed considering K = 2 to K = 10.

In order to describe genetic and geographically concordant groups of sampling locations we used spatial analysis of molecular variance (SAMOVA) [56]. This was performed based on genetic pairwise differences between sequences and geographic coordinates of sampling locations, through 100 annealing processes and 1,000 permutations using the SAMOVA software [56]. Different numbers of groups were tested (K = 2 to K = 10) and the F_SC_, F_ST_ and F_CT_ indices were plotted to investigate the number of groups (K) that best represent the spatial distribution of genetic variability.

Mantel tests [57] were performed using the mitochondrial and nuclear datasets to detect signals of isolation by distance (IBD). These tests were carried out considering a genetic distance matrix, constructed based on Slatkin’s linearized F_ST_'s [58], and a geographic distance matrix, that contains linear distances in kilometers between each pair of locations. Tests were performed using the Isolation By Distance Web Service (IBDWS) 3.23 [59] through 10,000 randomizations.

### Demographic analyses

Tajima’s D [60], Fu’s Fs [61], Fu and Li’s D* and F* [62] summary statistics were estimated after defining the best grouping configuration of sampling locations to investigate deviations from neutrality of NWS fly samples. For all tests, values near zero indicate that population sizes are stable, significant negative values indicate an excess of low frequency variants which are associated with positive selection or a demographic scenario of population expansion, and positive values indicate that a population has an excess of intermediary frequency variants resulting from the action of a balancing selection or population bottleneck [60–62]. Tests were performed for the concatenated mitochondrial sequences (CR, COI, COII) and each part of the *ChαE7* gene separately (introns iII and iIII, exons eIII and eIV) using DnaSP 5.0 [52]. The significance of Fu’s Fs was obtained by coalescence, with 1,000 replicates and a 95% confidence interval, and two tailed tests were used to determine the statistical significances of the other three tests.

Mismatch distribution analysis based on the distribution of pairwise differences between sequences was performed to better understand the demographic scenario of NWS fly samples. A unimodal distribution indicates recent expansion, while a multimodal distribution indicates population contraction, structure, influence of migration or subdivision [63–69]. This analysis was performed using the Arlequin 3.5 software [53], with the significance obtained by 10,000 permutations. The sum of the square deviations (SSD) and the *Raggedness* index (*rg*) were used to measure the adjustment to a population expansion model.

### Positive selection

The investigation of *ChαE7* positive selection signals was done based on the population structure of the sampling locations.

The frequencies of polymorphic nucleotides in the two codons associated to insecticide resistance (Gly137Asp and Trp251Leu) and the Hardy-Weinberg equilibrium between these were obtained with the Arlequin 3.5 software [53].

Fay and Wu’s H neutrality test [70] was performed using the DnaSP 5.0 software [52]. The orthologous sequence of *D*. *melanogaster* (GenBank accession number: NM079537) was used as the outgroup and statistical significance was accessed by coalescence through 1000 replicates, considering a confidence interval of 95%.

## Results

### Carboxylesterase E3 (*ChαE7*) Gene Characterization

*ChαE7* sequences from five individuals were obtained (GenBank accession numbers: KR067547- KR067551), although two of these were partial due to the absence of the initial sequence of intron iII. Sequences were approximately 3 Kb, comprising 111 bp of exon eII, around 2000 bp of intron iII (variable sizes due to presence of indels), 150 bp of exon eIII, 63 bp of intron iIII and 318 bp of exon eIV (Fig 1). Although the NWS fly eII, eIII, iIII and eIV regions have been described previously [41], this is the first report of the intron iII sequence. The two mutations associated with insecticide resistance, Gly137Asp and Trp251Leu, are located in exons eIII and eIV, respectively.

Nucleotide composition of exons (eIII and eIV) and introns (iII and iIII) of the five NWS fly individuals and the other Muscomorpha species (*L*. *cuprina*, *D*. *melanogaster* and *M*. *domestica*) is presented in Table 2. For introns, *M*. *domestica* was excluded due to absence of sequence.

**Table 2:** Nucleotide composition of *ChαE7* sequences from five NWS fly individuals and three Muscomorpha species.

Gene Region	Sample/Species	Nucleotide Composition (%)							
		A	C	T	G	A+T	G+C	AT skew	GC skew
**exon eIII**	BrCR20	29.33	13.33	36.67	20.67	66.00	34.00	-0.11	0.22
	Ch22	29.33	12.67	37.33	20.67	66.67	33.33	-0.12	0.24
	UyPa24	31.33	13.33	36.67	18.67	68.00	32.00	-0.08	0.17
	UyPa12	30.00	14.00	36.67	19.33	66.67	33.33	-0.01	0.16
	UySa21	30.00	14.67	36.00	19.33	66.00	34.00	-0.09	0.14
	Lucilia cuprina*	29.33	14.67	35.33	20.67	64.67	35.33	-0.09	0.17
	*Drosophila melanogaster*	22.00	20.67	26.00	31.33	48.00	52.00	-0.08	0.21
	*Musca domestica*	23.33	19.33	32.00	25.33	55.33	44.67	-0.16	0.13
**exon eIV**	BrCR20	27.36	16.67	33.65	22.33	61.01	38.99	-0.01	0.15
	Ch22	27.36	16.67	33.65	22.33	61.01	38.99	-0.01	0.15
	UyPa24	27.36	17.30	32.70	22.64	60.06	39.94	-0.09	0.13
	UyPa12	27.99	16.67	32.70	22.64	60.69	39.31	-0.08	0.15
	UySa21	27.36	17.30	32.70	22.64	60.06	39.94	-0.09	0.13
	Lucilia cuprina*	27.04	21.70	29.25	22.01	56.29	43.71	-0.04	0.01
	*Drosophila melanogaster*	24.92	26.48	22.43	26.17	47.35	52.65	0.05	-0.01
	*Musca domestica*	27.36	22.64	24.53	25.47	51.89	48.11	0.05	0.06
**intron iII**	BrCR20	34.49	14.51	35.06	15.95	69.55	30.45	-0.01	0.05
	Ch22	34.49	14.51	35.06	15.95	69.55	30.45	-0.01	0.05
	UyPa24	34.12	14.38	36.04	15.46	70.16	29.84	-0.03	0.04
	UyPa12	33.26	16.67	34.40	15.67	67.66	32.34	-0.02	-0.03
	UySa21	33.63	16.56	33.99	15.82	67.62	32.38	-0.01	-0.02
	Lucilia cuprina**	32.22	4.44	47.41	15.93	79.63	20.37	-0.19	0.56
	*Drosophila melanogaster*	34.52	11.90	41.67	11.90	76.19	23.81	-0.09	0.00
**intron iIII**	BrCR20	34.92	12.70	42.86	9.52	77.78	22.22	-0.01	-0.14
	Ch22	34.92	12.70	42.86	9.52	77.78	22.22	-0.01	-0.14
	UyPa24	38.10	11.11	42.86	7.94	80.95	19.05	-0.06	-0.17
	UyPa12	38.10	11.11	42.86	7.94	80.95	19.05	-0.06	-0.17
	UySa21	36.51	12.70	42.86	7.94	79.37	20.63	-0.08	-0.23
	Lucilia cuprina**	37.04	11.11	48.15	3.70	85.19	14.81	-0.13	-0.50
	*Drosophila melanogaster*	29.82	19.30	35.09	15.79	64.91	35.09	-0.08	-0.01

BrCR20, Ch22, UyPa12, UyPa24 and UySa21 indicate the five *C*. *hominivorax* individuals. Exons (eIII and eIV) and introns (iII and iIII) are considered separately.

* *L*. *cuprina* GenBank accession number U56636.

** *L*. *cuprina* GenBank accession number AY691508.

A high A+T content is observed in both exons and introns of NWS fly individuals, with minimum and maximum values of 60.06% and 80.95%. This was related to a higher proportion of T than A, indicated by the negative AT-skew. The GC-skew shows positive values for all individuals when exons were considered (indicating more Gs than Cs), while this was negative for most of the individuals in the case of the introns. However, both AT-skew and GC-skew were near to zero for intron iII (0.01–0.05), indicating that A and T nucleotides, as well as G and C, occurred in similar proportions.

*L*. *cuprina* had a more similar pattern to the NWS fly, consistent with their closer phylogenetic relationship, with an A+T content of 64.67% and 56.29% for eIII and eIV exons, respectively. The AT-skew was negative and the GC-skew positive for both exons of *L*. *cuprina*. However, the values were near to zero for exon eIV (-0.04 and 0.01).

*D*. *melanogaster* and *M*. *domestica* showed a more balanced distribution of nucleotide frequencies. However, *D*. *melanogaster* also had a high A+T content for intron iIII and iII sequences with values of 64.91 and 76.19%, respectively.

### Genetic diversity of populations

The CR, COI and COII fragment lengths were 488, 731 and 511 bp after alignment, defining 59, 44 and 35 haplotypes, respectively. Among these haplotypes, 10 of CR, 5 of COI and 1 of COII sequences were novel. The GenBank accession numbers are KR067531—KR067540 for the CR, KR067541—KR067545 for COI and KR067546 for COII. The mean genetic distance among haplotypes (uncorrected p-distance) was 0.005 for CR (range 0–0.015), 0.003 for COI (range 0–0.010) and 0.004 for COII (range 0–0.012). Next the CR, COI and COII sequences were concatenated in a 1726 bp fragment, defining 96 haplotypes that had been found previously [38] and 19 that were novel (haplotypes 283 to 301). As described previously [38], haplotype 140 was the most frequent (38/201), followed by haplotypes 78 (10/201) and 187 (7/201) (Table 3). The mean genetic distance (uncorrected p-distance) for the concatenated haplotypes was 0.004 (range 0–0.011).

**Table 3:** General diversity indices for mitochondrial CR, COI, COII and *ChαE7* data in NWS fly sampling locations.

ID	Concatenated CR,COI and COII				*ChαE7*			
	Nh	Haplotypes (no. of individuals)	Ĥ	π	Nh*	Haplotypes (no. of individuals)	Ĥ*	π
BTO	6	53(2), 64(2), **78**(1), **140**(2), 159(1), 166(1)	0.9167	0.0050	3	**2**(8), **3**(8), 15(2)	0.6275	0.0164
BGN	9	43(2), 59(1), 66(1), 80(1), 96(1), 134(1), 165(1), 286(1), 287(1)	0.9778	0.0055	3	**1**(2), **2**(15), **3**(3)	0.4263	0.0123
BGO	6	1(2), 111(1), 185(2), 191(1), 283(3), 288(1)	0.8889	0.0043	2	**2**(18), **3**(2)	0.1895	0.0052
BCA	7	1(1), 51(1), **140**(4), 150(1), 155(1), 169(1), 183(1)	0.8667	0.0032	4	**1**(3), **2**(14), **3**(2), 25(1)	0.5000	0.0144
BCR	5	120(1), 125(1), **140**(2), 143(5), 146(1)	0.7556	0.0015	6	**1**(6), **2**(2), **3**(9), 8(1), 26(1), 27(1)	0.7263	0.0155
BAQ	7	112(1), **140**(2), 141(2), 142(1), 189(1), 284(2), 289(1)	0.9333	0.0036	7	**1**(4), **2**(2), 4(4), 7(4), 16(2), 17(2), 18(2)	0.8842	0.0206
BSS	5	41(2), 118(1), **140**(4), **187**(2), 190(1)	0.8222	0.0038	3	**2**(16), **3**(2), 13(2)	0.3579	0.0055
BES	7	15(1), 50(1), 77(1), 103(1), **140**(2), **187**(2), 192(1)	0.9444	0.0044	5	**1**(3), **2**(10), **3**(3), 8(1), 28(1)	0.6667	0.0181
BCI	9	51(1), **140**(3), 147(1), 154(1), 169(1), **187**(2), 193(1), 290(1), 291(1)	0.9394	0.0040	5	**1**(10), **2**(8), **3**(4), 5(1), 6(1)	0.7138	0.0199
BFV	8	14 (2), 19(1), 23(1), **78**(1), 82(1), **140**(1), 145(1), 292(1)	0.9722	0.0040	4	**1**(4), **2**(10), 11(2), 12(2)	0.6536	0.0165
BSA	10	21(1), 65(1), 67(1), 83(1), 115(1), **140**(1), 170(1), 171(1), 178(1), 293(1)	1	0.0043	3	**1**(15), **3**(4), 31(1)	0.4158	0.0079
BPM	8	**78**(2), 85(1), 100(1), 122(1), 139(1), **140**(1), 186(1), 188(3)	0.9273	0.0046	7	**1**(13),**2**(2), **3**(1), 5(2), 14(2), 29(1), 30(1)	0.6494	0.0164
PYB	8	9(1), 49(1), 63(1), **78**(2), 137(1), **140**(2), 167(1), 188(1)	0.9556	0.0043	6	**1**(8), **2**(7), **3**(2), 4(1), 32(1), 33(1)	0.7368	0.0197
UPM	6	75(1), **78**(1), 99(1), **140**(5), 296(1), 297(1)	0.7778	0.0041	8	**1**(8), **2**(2), **3**(3), 21(2), 22(2), 44(1), 45(1), 46(1)	0.8211	0.0202
UST	8	42(1), 50(2), 81(1), 114(1), **140**(2), **187**(1), 294(1), 295(1)	0.9556	0.0043	8	**1**(10), **2**(1), **3**(1), 6(3), 23(2), 47(1), 48(1), 49(1)	0.7421	0.0110
UDA	5	105(1), 119(1), **140**(2), 152(2), 298(1)	0.9048	0.0029	5	**1**(6), **3**(1), 4(2), 20(2), 24(2)	0.7912	0.0178
UBM	8	25(1), 31(1), 74(1), **78**(2), 86(1), 111(1), **140**(2), 176(2)	0.9455	0.0049	4	**1**(17), **2**(3), 5(1), 36(1)	0.3983	0.0128
UCC	9	27(2), 43(1), 97(1), 108(1), 113(1), 136(1), **140**(1), 162(1), 177(1)	0.9778	0.0054	6	**1**(7), **2**(8), **3**(2), 8(1), 42(1), 43(1)	0.7368	0.0193
UCO	7	16(1), **140**(2), 179(2), 185(1), 285(2), 299(1), 300(1)	0.9333	0.0055	9	**1**(6), **2**(5), **3**(3), 5(1), 37(1), 38(1), 39(1), 40(1), 41(1)	0.8526	0.0209
UJS	7	5(1), 6(1), 71(3), 72(1), 121(1), 128(1), 301(1)	0.9167	0.0047	6	**1**(8), 9(3), 10(3), 19(2), 34(1), 35(1)	0.7712	0.0182
APL	3	52(1), 68(2), **78**(1)	0.8333	0.0027	6	**1**(3), **2**(1), **3**(1), 4(1), 5(1), 6(1)	0.8929	0.0227

Most frequent haplotypes are in bold. ID = site identification code; Nh = number of haplotypes; Nh* = number of phased haploid sequences; Ĥ = haplotipic diversity; Ĥ* = expected heterozygosity; π = nucleotidic diversity.

Intron iII, exon eIII, intron iIII and exon eIV of the *ChαE7* gene were 157, 150, 63 and 282 bp length, respectively, resulting in a total sequence length of 652 bp. Based on this, we defined 49 haplotypes (GenBank accession numbers: KR067482—KR067530). Haplotypes 1 (133/402), 2 (132/402) and 3 (52/402) were the most frequent (Table 3). The mean uncorrected genetic p-distance among the haplotypes was 0.019 (range 0–0.034).

Haplotype distribution in sampling locations, nucleotide diversity (π) and haplotype diversity/expected heterozygosity (Ĥ) estimated from concatenated mitochondrial and *ChαE7* gene sequences are shown in Table 3. Low nucleotide diversity was observed for both mitochondrial and nuclear genes, ranging from 0.0015 to 0.0055 in mitochondrial and 0.0052 to 0.0227 in the *ChαE7* datasets. However, high haplotype diversity was found, ranging from 0.7556 to 1 and 0.1895 to 0.8929 in the mitochondrial and *ChαE7* datasets, respectively.

### Structure analyses

The non-hierarchical AMOVA, considering all sampled locations as a unique population, showed higher structure when considering the *ChαE7* gene, with fixation indexes (Φ_ST_) of 0.235 and 0.094 for the *ChαE7* and mitochondrial datasets respectively (Table 4).

**Table 4:** AMOVA results for mitochondrial (CR, COI and COII) and *ChαE7* genes.

	CR, COI and COII	*ChαE7*
**Variation among populations**	9.43	23.51
**Variation within populations**	90.57	76.49
**Ф**_**ST**_	0.094 (P< 0.001)	0.235 (P< 0.001)

Ф_ST_: *Phi*-_ST_ parameter (fixation index).

Pairwise F_ST_ estimates with the *ChαE7* gene showed significant values between approximately 65% of all locations pairs (137/210) (S1 Table), decreasing to 33% (70/210) when considering mitochondrial data (S2 Table). In the latter case, BCR, BSA and APL were the locations which had the most significant values (18/20, 14/20 and 12/20, respectively).

Examination of the mitochondrial data showed that the NWS fly populations were genetically and geographically homogeneous, according to DAPC and SAMOVA analyses. In addition, application of the Mantel test showed no correlation between geographic and genetic distances (r = 0.0445, p > 0.05; S1 Fig), indicating an absence of isolation-by-distance and no limited gene flow among NWS fly sampled locations. Therefore, all locations analyzed were considered as belonging to a unique group (K = 1) based on the mitochondrial data.

**Fig 2:**
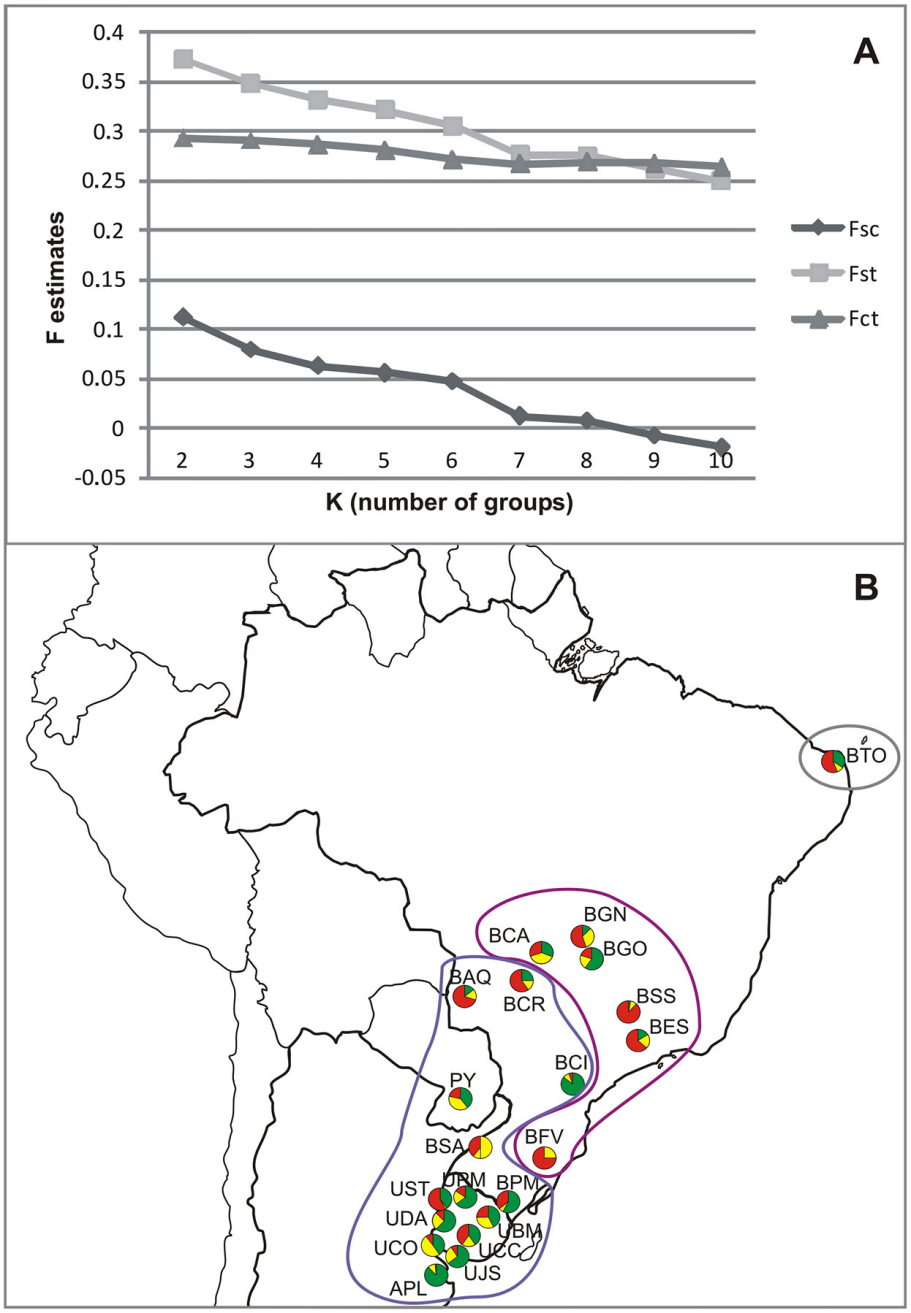
Structure analyses results for esterase data (K = 3). (A) SAMOVA F indices. F_CT_: differentiation between groups; F_SC_: differentiation between sampling locations within groups; F_ST_: differentiation between sites between groups. (B) Pie charts of the posterior probability to be from a group estimated by DAPC (i.e. Red, Yellow and Green) and SAMOVA grouping (i.e. I, II and III). SAMOVA groups (I, II and III), considered for posterior analyses, are defined by the grey, purple and blue contours.

In contrast, an increased degree of genetic structure was detectable based on the carboxylesterase E3 (*ChαE7*) gene according to DAPC and SAMOVA analyses, with K = 3 as the most probable geographic arrangement (Fig 2). SAMOVA indicated that K = 3 was the most probable scenario due to high F_CT_ and low F_SC_ values (Fig 2A). A North-South pattern was observed based on the DAPC results, with a higher frequency of individuals belonging to group Red and group Green in higher and lower latitudes, respectively (Fig 2B). Group Yellow was more frequent in some southern locations, such as BSA and UCO. The three genetically and geographically distinct groups, based on both analyses, were: I) BTO; II) BGN, BGO, BCA, BSS, BES, BFV and III) BCR, BAQ, BCI, PYB, BSA, UST, UPM, UDA, BPM, UBM, UCC, UCO, UJS, APL (Fig 2B). Mantel testing indicated no correlation between geographic and genetic distances (r = 0.2458, p = 0.05) (S1 Fig), with absence of isolation-by-distance among NWS fly-sampled locations.

### Demographic inference

**Fig 3:**
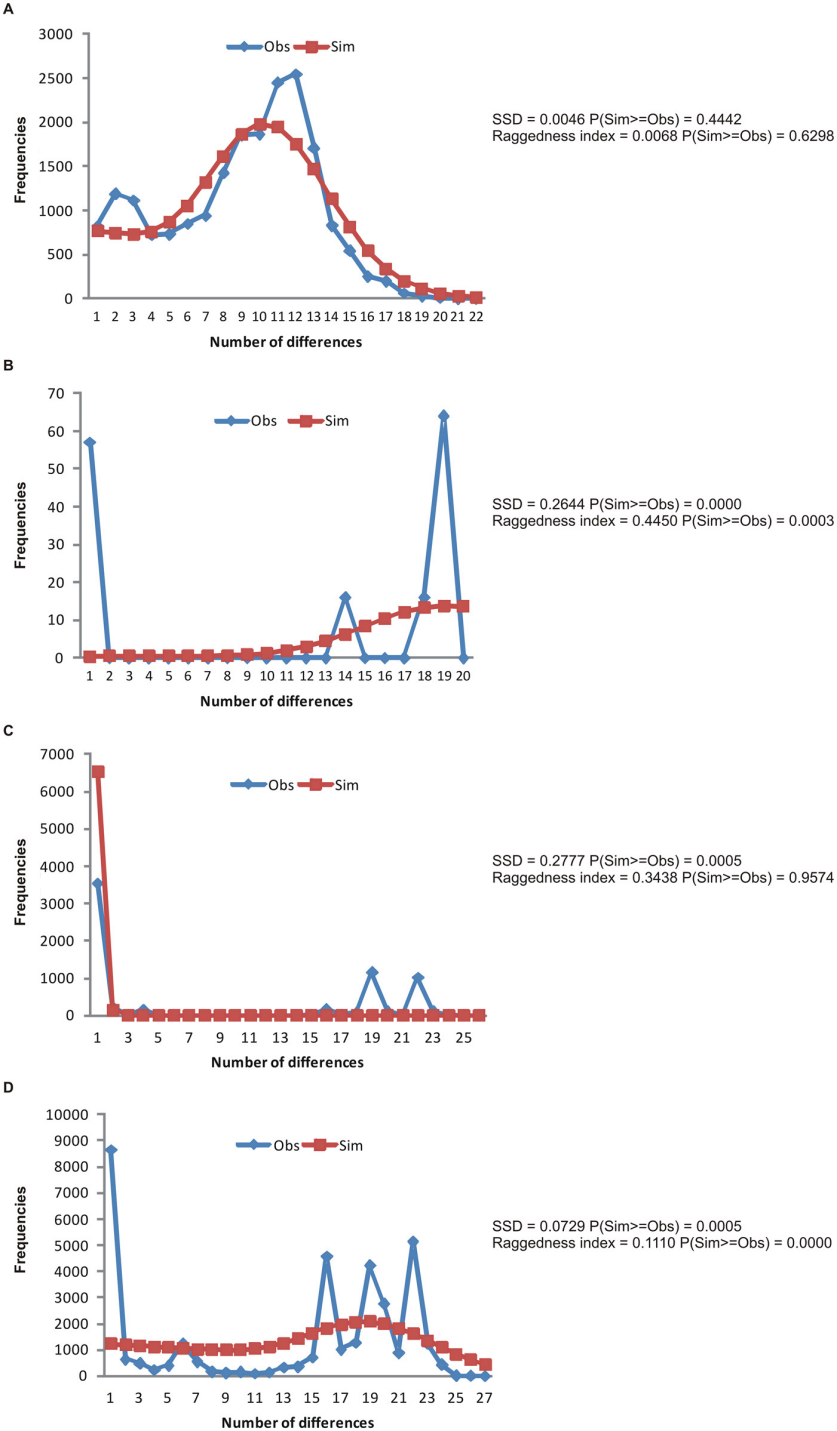
Mismatch distribution analyses results. (A) Mitochondrial sequences considering the complete sample in a group. (B) *ChαE7* sequences considering Group I. (C) *ChαE7* sequences considering Group II. (D) *ChαE7* sequences considering Group III from SAMOVA, respectively.

The demographic history of NWS fly was inferred based on the groups defined by DAPC and SAMOVA, considering one group for mitochondrial data and three for the *ChαE7* gene. Tajima’s D, Fu’s Fs and Fu & Li’s D* and F* summary statistics yielded significant negative values for the mitochondrial data, while no significant results were found for the *ChαE7* gene, except the Tajima’s D and Fu & Li’s F* for the intron iII in group I (Table 5). Mismatch distribution analysis resulted in a unimodal distribution of pairwise differences between mitochondrial sequences, adjusted to the theoretical distribution of a demographic scenario of recent population expansion (P [SSD] = 0.4442, P [raggedness] = 0.6298). This analysis resulted in multimodal distributions for the three groups of populations with significant values of SSD and raggedness index for the *ChαE7* gene, rejecting the null hypothesis of population expansion (Fig 3).

**Table 5:** Neutrality tests for mitochondrial (MIT) and *ChαE7* sequence data.

Marker	Group	Gene Part	Tajima's D	Fu's Fs	Fu and Li's D*	Fu and Li's F*
MIT	1 group	CR,COI,COII	-1,90249	-127,565	-4,81061	-4,13967
*ChαE7*	**Group I**	intron iII	**2,18639**	5,10400	1,30779	**1,78900**
	**Group I**	intron iIII	1,21656	1,66400	1,02360	1,23379
	**Group I**	exon eIII	1,44464	3,85800	1,25898	1,50893
	**Group I**	exon eIV	1,84421	3,52600	1,19899	1,58319
	**Group II**	intron iII	0,89852	7,66100	1,46209	1,50027
	**Group II**	intron iIII	1,51772	3,65300	0,67407	1,09669
	**Group II**	exon eIII	1,10336	4,42200	1,02433	1,24009
	**Group II**	exon eIV	0,90966	1,34100	0,13287	0,46537
	**Group III**	intron iII	0,76406	0,449	-1,70743	-0,82276
	**Group III**	intron iIII	-0,35839	-2,62800	1,30897	0,83630
	**Group III**	exon eIII	0,12338	-4,53300	-1,48298	-1,05786
	**Group III**	exon eIV	-0,48626	-2,91300	0,98188	0,49412

Statistically significant values are in bold.

### Positive selection

From a total of 49 alleles, 31 had a glycine and 18 had an aspartate in position 137, while 43 had a tryptophan, 5 a serine and 1 a leucine in position 251 (S3 Table).

The presence of glycine (coded by GGG or GGC) and aspartate (coded by GAC or GAT) in position 137 are associated, respectively, with susceptibility and resistance to diethyl-organophosphates in dipterous species. The presence of tryptophan in position 251 (coded by TGG) defines the susceptible genotype, while serine and leucine (coded by TCG and TTG, respectively) are associated with dimethyl-organophosphates resistance. The substitution of the amino acid tryptophan by a leucine in position 251 was associated previously with dimethyl-organophosphates resistance in *L*. *cuprina* [24]. However, another study found that dimethyl-organophosphate resistant lineages of *M*. *domestica* have a serine substitution in place of a tryptophan [71], suggesting that substitution with small residues, such as leucine, glycine or serine could be the factor that leads to the increase of insecticide hydrolysis by carboxylesterase E3. No alleles with residues predicted to confer resistance in both sites positions were observed (i.e. aspartate in position 137; serine or leucine in position 251).

Residue combinations in these two positions for 25 susceptible alleles and 24 resistant alleles did not reflect the real frequency of susceptibility and resistance in field populations. To explore this issue further, we specifically analyzed the two residues associated with insecticide resistance at the nucleotide level and considering allele frequencies taking into account the three groups for the *ChαE7* gene.

**Fig 4:**
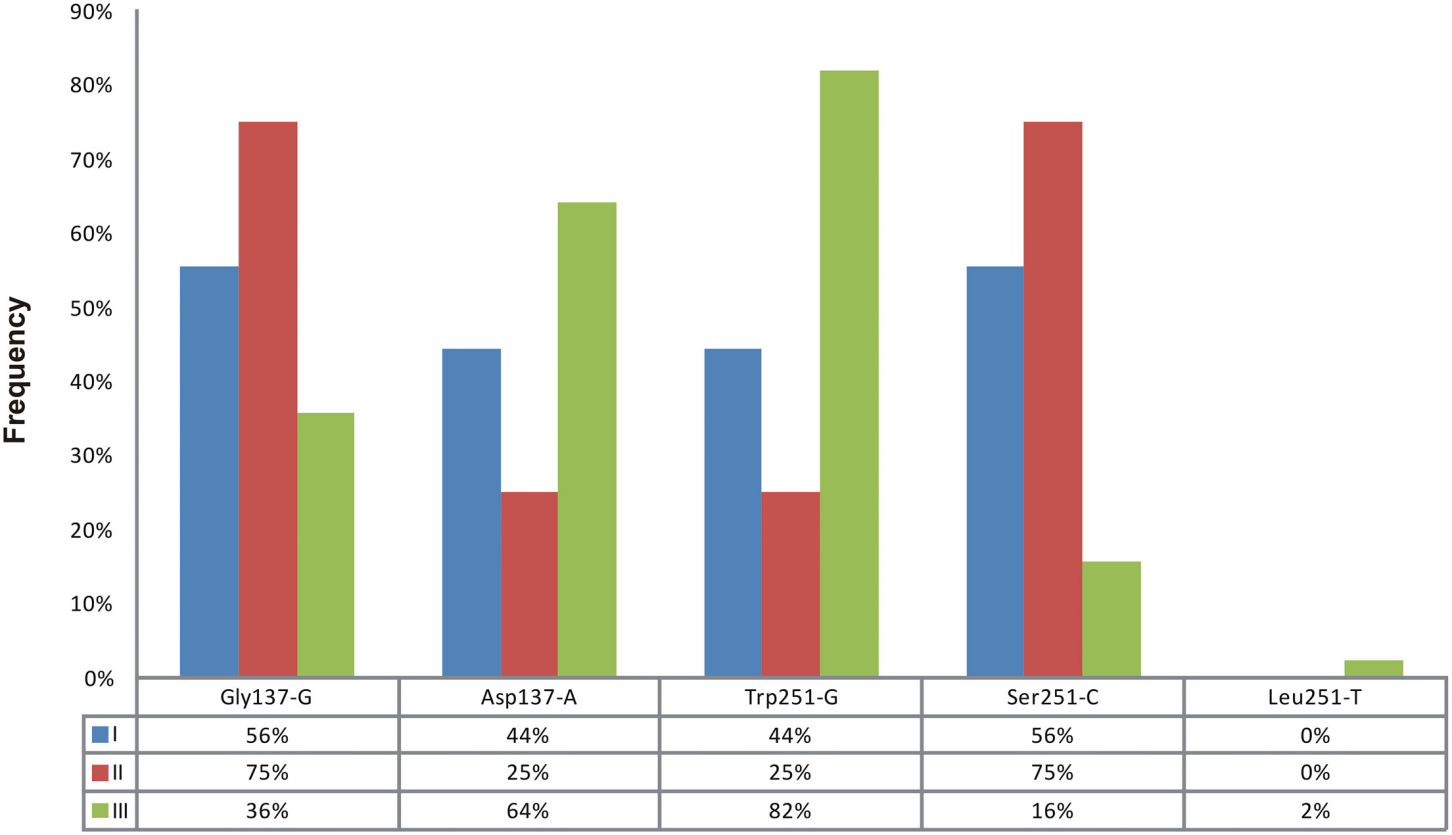
Frequencies of polymorphic nucleotides related to the determination of susceptibility and resistance in the codons 137 and 251 of the *ChαE7* sequence. The structure in groups I, II and III (K = 3) was analyzed. Codons were named for the encoded amino acid, *ChαE7* sequence position (amino acid number) and the nucleotide substitution of that codon (Gly137-G, Asp137-A, Trp251-G, Leu251-T and Ser251-C).

Considering codon 137, the nucleotide present in the second position defines the susceptibility (guanine; named Gly137-G) or resistance (adenine; named Asp137-A). Similarly, the nucleotide present in the second position of codon 251 defines susceptibility (guanine; named Trp251-G) and the two resistant types (thymine and cytosine; named Leu251-T and Ser251-C, respectively). The frequencies of each nucleotide in these two codon positions for each group of sampling locations are showed in Fig 4.

Group I (consisting only of BTO) showed the same frequencies of Gly137-G and Ser251-C (56%), and Asp137-A and Trp251-G (44%). Group II showed the same pattern of complementary frequencies for the Gly137-G and Ser251-C (75%) and for Asp137-A and Trp251-G (25%). In group III, the frequency of Asp137-A was 64% and that of Trp251-G was 82%, while the frequencies of Gly137-G and Ser251-C were 36% and 16%, respectively. This showed that there was a higher frequency of individuals in groups I and II with a genotype conferring resistance to dimethyl-organophosphates, while there was a higher frequency of diethyl-organophosphates resistant flies in group III. Another difference between these groups is the low frequency (less than 3%) of Leu251-T that appeared only in group III.

These two polymorphic codon positions (second codon positions from codons 137 and 251) were not in Hardy-Weinberg equilibrium in the three groups of sampled locations, as the observed heterozygosity was smaller than expected (Table 6).

**Table 6:** Hardy-Weinberg equilibrium tests for the two polymorphic codon positions related to the determination of susceptibility and resistance.

	codon 137 (2nd position)				codon 251 (2nd position)			
Groups	Ho	He	P-value	s.d.	Ho	He	P-value	s.d.
**I**	0.000	0.523	**0.003**	0.000	0.000	0.523	**0.003**	0.000
**II**	0.017	0.378	**0.000**	0.000	0.017	0.378	**0.000**	0.000
**III**	0.209	0.462	**0.000**	0.000	0.119	0.302	**0.000**	0.000

Statistically significant values are in bold. Ho = observed heterozygosity; He = expected heterozygosity; s.d. = standard deviation.

Application of Fay & Wu’s H neutrality test showed statistically significant negative values for the exons eIII and eIV in groups II and III, indicating positive selection (Table 7). Both exons showed no significant results in group I, indicating that there was no positive selection signature. Intron iIII showed no significant values for any group, although this was expected since introns normally undergo neutral evolution. However, this test was not performed for intron iII due to absence of the corresponding sequence from another species that gives a good and reliably alignment, which is a prerogative for the test.

**Table 7:** Fay and Wu’s H neutrality test for the *ChαE7* gene.

Grouping		Gene Part	Fay & Wu's H
**3 groups**	**I**	intron iII	-
		intron iIII	0.1569
		exon eIII	-2.8497
		exon eIV	-0.0523
	**II**	intron iII	-
		intron iIII	-0.5043
		exon eIII	**-3.1535**
		exon eIV	**-3.8918**
	**III**	intron iII	-
		intron iIII	0.1391
		exon eIII	**-4.4285**
		exon eIV	**-6.6635**

Statistically significant values are in bold.

## Discussion

Partial characterization of the carboxylesterase E3 *ChαE7* gene allowed its use as a nuclear molecular marker for comparisons with previously studied mitochondrial markers [38,39] and for investigation of the genetic and geographic structure, demographic history and selection pressure of NWS fly populations inhabiting the southern region of the Amazon river basin.

Based on the homologous *ChαE7* sequence from *L*. *cuprina* [27], we expected to generate a fragment of around 921 bp following PCR amplification, instead of the approximately 2600 bp obtained for the NWS fly. This difference was due to the increased length of intron iII in the NWS fly. *L*. *cuprina* has a length of only approximately 280 bp [27], which is almost seven times smaller than the observed length for NWS fly (around 2000 bp). As a further comparison, the length of intron iII in *D*. *melanogaster* is 85 bp [72] and that in *M*. *domestica* is 63 bp [26]. This indicates that the length of intron iII has high variability within Muscomorpha. In contrast, intron iIII showed a smaller variation in length in the same species, as this was 63 bp in the NWS fly, 56 bp in *L*. *cuprina* [27], 58 bp in *D*. *melanogaster* [72] and 62 bp in *M*. *domestica* [26]. Unlike introns, the sequences of exons eIII and eIV are conserved with respect to the length, with only the *D*. *melanogaster* exon eIV presenting three more base pairs in the region analyzed, totalizing 321bp.

The NWS fly *ChαE7* nucleotide composition was similar to that of *L*. *cuprina*, consistent with their closer phylogenetic relationship. Both species showed a bias toward A+T content, which was suggested previously to be a remarkable feature of the mitochondrial genome of Calliphoridae species [73,74]. In general, a negative AT-skew and a positive GC-skew was observed, indicating different nucleotides usage. The skewness is a measure of the relative number of As to Ts, in the case of AT-skew, and Gs to Cs, in the case of GC-skew [75].

The low genetic distance among haplotypes in both datasets reflects the low nucleotide diversity (π), although haplotype diversity (Ĥ) was high in both cases. This pattern of diversity is typically observed in a demographic scenario of recent populations expansion [76].

All population analyses pointed to an increased genetic structure of the carboxylesterase E3 *ChαE7* gene in relation to the mitochondrial gene sequences. This was indicated by the AMOVA Φ_ST_ value which was 2.5 times greater and a higher percentage of pairwise F_ST_ significant values for the *ChαE7* gene. In a similar manner, the DAPC and SAMOVA data showed the *ChαE7* genetic structure of NWS fly populations, revealing three geographically non-concordant groups (K = 3). There was also an apparent North-South pattern of variability distribution, which did not appear to result from isolation-by-distance (IBD), as indicated by the non-significant Mantel tests between genetic and geographic distances. A previous investigation suggested that IBD can lead to false positives in tests of population structure and detection of loci evolving under selection [77], although the present finding of no correlation between geographic and genetic distances matrices indicates that the population structure detected is not biased or confounded by IBD. In this scenario, the three *ChαE7* groups could result from selection because of regional differences of insecticide usage.

For mitochondrial data, the AMOVA Φ_ST_ value was low and the BCR, BSA and APL locations showed the highest percentage of pairwise F_ST_ significant values. BCR is located in Mato Grosso do Sul (MS), the central region of Brazil, while BSA is located in Rio Grande do Sul (RS), the southern region of Brazil. Both states are noted for high Brazilian cattle production, being the second and tenth in amount of slaughter in 2013, respectively [78]. Not all locations sampled in these two states showed an increased percentage of significant pairwise F_ST_ values, indicating an unusual pattern of both locations which could be linked to the central points for cattle interchange between places. APL is the unique population sampled in Argentina (located near southern Brazil), which is also a region of high cattle production. The results of the DAPC and SAMOVA analyses supported a panmixia scenario for the mitochondrial gene data, consistent with a previous study [38], and therefore did not reveal a genetic/geographic structure of the sampled locations.

These findings are similar to results from other insect pests. The codling moth *Cydia pomonella* exhibits a low level of genetic structure in four countries (France, Italy, Armenia and Chile) based on seven microsatellite loci, probably due to its high capacity of migration, while a higher population differentiation was detected with insecticide resistance markers [79]. In addition, population differences in resistance are larger than differences in microsatellite variation for the Australian diamondback moth (*Plutella xylostella*), without evidence of spatial structure for resistance through IBD [80].

Tajima’s D, Fu’s Fs and Fu & Li’s D* and F* significant negative values and unimodal distribution of pairwise differences indicate a history of population expansion according to mitochondrial gene data. However, the finding of no significant results for these neutrality tests and multimodal distribution of pairwise differences for the *ChαE7* gene indicates a different history which, in addition to the population analysis, supports the potential role of selection.

The results of Fay & Wu’s H [70] neutrality test indicated positive selection on both exons (eIII and eIV) from groups II and III but not for group I. The absence of a positive selection signal in the case of group I could be due to sampling size since it was composed of only nine individuals from one location (BTO). Resistant genotypes had a high frequency in all sampled areas (Fig 4), with dimethyl-organophosphate resistance represented more in lower latitudes and diethyl-organophosphate resistance was greater in higher latitudes. These differences of *ChαE7* allele frequencies could reflect the usage of different insecticide compounds in each geographic location. Alternatively, they could reflect differences in alleles frequencies of a *Modifier* locus, which has been related to a fitness compensation of the negative effects of both resistant mutations Gly137Asp and Trp251Leu (i.e. developmental instability) in *L*. *cuprina* [81–83].

High levels of insecticide resistance are also observed for *Aedes aegypti* in three Brazilian states (Rio de Janeiro, Espírito Santo and Ceará) [84,85] and for the house fly *Musca domestica* in three Argentinean populations [86]. The horn fly, *Haematobia irritans*, showed high frequencies of the *kdr* mutation, associated with high levels of pyrethroid resistance, in southern Chile [87]. Our results, in addition to many other studies, indicate that insecticide resistance is widespread throughout South America. It is likely that use of different compounds for control of distinct insect species has led to resistance through multiple mechanisms, which should be cause for great concern.

A negative association between the mutations in the carboxylesterase E3 *ChαE7* gene that confer resistance was detected, indicating that they are not present in the same allele. This was reported previously for *L*. *cuprina* [88] and NWS fly in four of the locations under investigation in the present study (UST, UDA, UBM and UCC) [89].

Taken together, the observed low heterozygosity in the two polymorphic codon positions associated with susceptibility and resistance, the negative association between nucleotides in the same codons and the significant negative Fay & Wu’s H values for exons suggest that the *ChαE7* gene may be undergoing evolution under positive selection. The present findings indicate that this could be due to the use of organophosphate compounds on NWS fly populations in South America. This is consistent with previous studies on the demographic history of this species [38,39]. Many recent population studies have investigated effects on mitochondrial and nuclear markers together, in attempts to understand the history of the species through genetic variation comparisons (for example, see [90,91]).

Livestock production began in the Americas around 500 years ago, resulting in the likely passive distribution and unclear geographic structure of this species and of some concordant insect pests. For example, approximately half of tick species have a genetic structure based mainly on the movement capacity of their respective hosts. Less mobile hosts typically support a low gene flow with highly structured tick populations. In contrast, highly mobile hosts allow a higher gene flow between ticks, leading to a low population structure [92]. As in the case of tick populations, the NWS fly has a notorious relation with its host during the larval stage and can be dispersed through host movement. However, the geographic structure of NWS fly populations has not been completely resolved and no clear boundaries between groups of populations have been identified. Genotyping-By-Sequencing (GBS) is a recent promising method that could be used to help achieve this aim, as this could lead to identification of hundreds or thousands of molecular markers (single nucleotide polymorphisms, SNPs) without the requirement of a completely sequenced genome [93].

When selecting a target region for a SIT based program, it is important to analyze the insecticide susceptibility of populations. Here, we have confirmed that the two mutations in the carboxylesterase E3 *ChαE7* gene associated with organophosphate resistance in many species, Gly137Asp and Trp251Leu, are also related to this mechanism in NWS fly through Darwinian selection. Additionally, we showed that NWS fly populations from SAG have high frequencies of organophosphate resistant genotypes, which can represent a barrier for the success of a control program based on SIT.

## Conclusions

The genetic data presented here showed that at least three groups of NWS fly populations can be found in SAG, but with no clear geographic correlation, and resistance to organophosphate insecticides is widespread and found in high frequency in most of the analyzed locations. These aspects may represent barriers to implement a successful control program based on SIT. New studies based on dense SNP genotyping will be necessary in order to refine previous results of population structure and to identify additional molecular markers that could have evolved under positive selection. This will allow the detection and monitoring of other genes involved in insecticide resistance and their frequencies in natural populations.

## Supporting Information

S1 Fig Mantel test between geographic and genetic distances to detect isolation by distance. A) Mitochondrial data; B) carboxylesterase E3 (*ChαE7*) gene.
(TIF)

S1 Table Pairwise F_ST_ estimates from carboxylesterase E3 (*ChαE7*) data. Statistically significant values are in bold.
(DOCX)

S2 Table Pairwise F_ST_ estimates from mitochondrial data. Statistically significant values are in bold.
(DOCX)

S3 Table Amino acids residues observed in positions 137 and 251 for 49 haplotypes of carboxylesterase E3 (*ChαE7*) obtained from field samples. Additionally, mitochondrial haplotypes related to each carboxylesterase E3 (*ChαE7*) haplotype are shown. E3 hap: carboxylesterase E3 haplotypes; Mit hap: mitochondrial haplotypes.
(DOCX)
