# Supplementary figures and images for: Incongruent Nuclear and Mitochondrial Genetic Structure of New World Screwworm Fly Populations Due to Positive Selection of Mutations Associated with Dimethyl- and Diethyl-Organophosphates Resistance
Source: PLoS One. 2015 Jun 1;10(6):e0128441. doi: 10.1371/journal.pone.0128441 (PMC4451984; doi:10.1371/journal.pone.0128441)

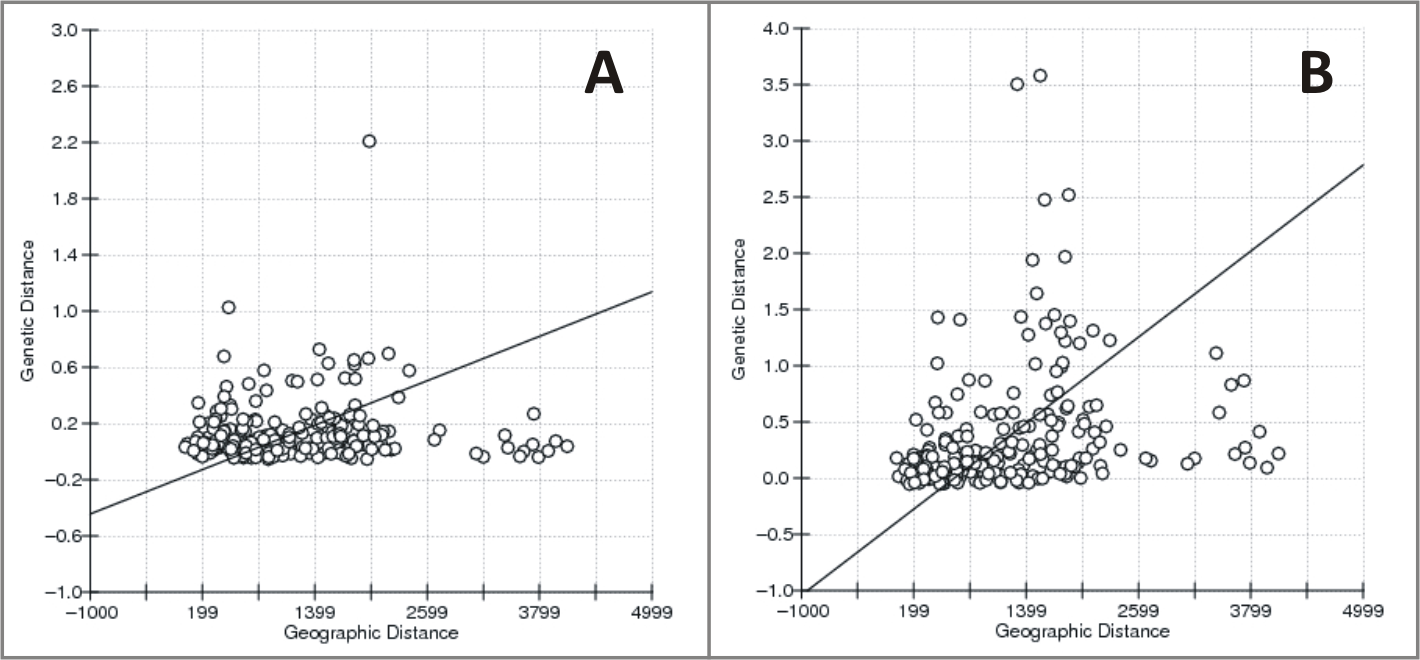

Supplement: S1 Fig — A) Mitochondrial data; B) carboxylesterase E3 (ChαE7) gene. (TIF) [file pone.0128441.s001.tif]
