# Supplementary material for: Incongruent Nuclear and Mitochondrial Genetic Structure of New World Screwworm Fly Populations Due to Positive Selection of Mutations Associated with Dimethyl- and Diethyl-Organophosphates Resistance
Source: PLoS One. 2015 Jun 1;10(6):e0128441. doi: 10.1371/journal.pone.0128441 (PMC4451984; doi:10.1371/journal.pone.0128441)
Supplement: S1 Table — Statistically significant values are in bold. (DOCX) [file pone.0128441.s002.docx]

**S1 Table. Pairwise F_ST_ estimates from carboxylesterase E3 (*ChαE7*) data.**

|  | **BTO** | **BGN** | **BGO** | **BCA** | **BCR** | **BAQ** | **BSS** | **BES** | **BCI** | **PYB** | **BFV** | **BSA** | **UST** | **UPM** | **UDA** | **BPM** | **UBM** | **UCC** | **UCO** | **UJS** | **APL** |
| --- | --- | --- | --- | --- | --- | --- | --- | --- | --- | --- | --- | --- | --- | --- | --- | --- | --- | --- | --- | --- | --- |
| **BTO** | 0.0000 |  |  |  |  |  |  |  |  |  |  |  |  |  |  |  |  |  |  |  |  |
| **BGN** | **0.1231** | 0.0000 |  |  |  |  |  |  |  |  |  |  |  |  |  |  |  |  |  |  |  |
| **BGO** | **0.2925** | 0.0152 | 0.0000 |  |  |  |  |  |  |  |  |  |  |  |  |  |  |  |  |  |  |
| **BCA** | 0.0985 | -0.0451 | 0.0580 | 0.0000 |  |  |  |  |  |  |  |  |  |  |  |  |  |  |  |  |  |
| **BCR** | **0.2006** | **0.4020** | **0.5883** | **0.3419** | 0.0000 |  |  |  |  |  |  |  |  |  |  |  |  |  |  |  |  |
| **BAQ** | **0.1339** | **0.3040** | **0.4664** | **0.2595** | **0.1241** | 0.0000 |  |  |  |  |  |  |  |  |  |  |  |  |  |  |  |
| **BSS** | **0.2898** | 0.0158 | -0.0495 | 0.0580 | **0.5851** | **0.4637** | 0.0000 |  |  |  |  |  |  |  |  |  |  |  |  |  |  |
| **BES** | 0.0400 | 0.0100 | **0.1747** | -0.0227 | **0.1967** | **0.1586** | **0.1732** | 0.0000 |  |  |  |  |  |  |  |  |  |  |  |  |  |
| **BCI** | **0.1497** | **0.1909** | **0.3701** | **0.1328** | **0.0766** | **0.1260** | **0.3680** | 0.0315 | 0.0000 |  |  |  |  |  |  |  |  |  |  |  |  |
| **PYB** | **0.1490** | **0.1787** | **0.3680** | **0.1192** | **0.0904** | **0.1153** | **0.3655** | 0.0206 | -0.0423 | 0.0000 |  |  |  |  |  |  |  |  |  |  |  |
| **BFV** | **0.1131** | -0.0217 | **0.1050** | -0.0419 | **0.3033** | **0.2362** | **0.1042** | -0.0319 | 0.0866 | 0.0718 | 0.0000 |  |  |  |  |  |  |  |  |  |  |
| **BSA** | **0.5264** | **0.6216** | **0.7817** | **0.5603** | **0.1916** | **0.3606** | **0.7780** | **0.4309** | **0.1896** | **0.2128** | **0.5052** | 0.0000 |  |  |  |  |  |  |  |  |  |
| **UST** | **0.4645** | **0.5494** | **0.7158** | **0.4873** | **0.1950** | **0.3080** | **0.7123** | **0.3611** | **0.1350** | **0.1584** | **0.4272** | 0.0462 | 0.0000 |  |  |  |  |  |  |  |  |
| **UPM** | **0.1761** | **0.3299** | **0.5065** | **0.2722** | 0.0183 | **0.0691** | **0.5039** | **0.1457** | 0.0299 | 0.0371 | **0.2288** | **0.1547** | **0.1176** | 0.0000 |  |  |  |  |  |  |  |
| **UDA** | **0.2131** | **0.3825** | **0.5827** | **0.3183** | -0.0046 | 0.0332 | **0.5788** | **0.1759** | 0.0468 | 0.0456 | **0.2717** | **0.1656** | **0.1519** | -0.0186 | 0.0000 |  |  |  |  |  |  |
| **BPM** | **0.3682** | **0.4246** | **0.5921** | **0.3616** | **0.1571** | **0.2275** | **0.5892** | **0.2423** | 0.0508 | 0.0620 | **0.3001** | 0.0610 | 0.0289 | **0.0695** | **0.0857** | 0.0000 |  |  |  |  |  |
| **UBM** | **0.4536** | **0.4974** | **0.6631** | **0.4333** | **0.2286** | **0.3160** | **0.6599** | **0.3148** | **0.0985** | **0.1120** | **0.3684** | 0.0556 | 0.0424 | **0.1387** | **0.1640** | -0.0225 | 0.0000 |  |  |  |  |
| **UCC** | **0.1194** | **0.1501** | **0.3418** | 0.0923 | 0.0890 | **0.1295** | **0.3394** | -0.00317 | -0.0396 | -0.0446 | 0.0511 | **0.2464** | **0.1959** | 0.0485 | 0.0633 | **0.0930** | **0.1480** | 0.0000 |  |  |  |
| **UCO** | 0.0848 | **0.2069** | **0.3932** | **0.1513** | 0.0143 | 0.0567 | **0.3908** | 0.0370 | -0.0218 | -0.0177 | **0.1165** | **0.2129** | **0.1676** | -0.0099 | -0.0020 | **0.0900** | **0.1598** | -0.0209 | 0.0000 |  |  |
| **UJS** | **0.2923** | **0.3880** | **0.5675** | **0.3257** | **0.0918** | **0.1042** | **0.5643** | **0.1997** | 0.0460 | 0.0383 | **0.2705** | **0.1159** | **0.0913** | 0.0194 | -0.0033 | 0.0171 | 0.0753 | 0.0721 | 0.0447 | 0.0000 |  |
| **APL** | **0.1791** | **0.3150** | **0.5506** | **0.2421** | 0.0011 | 0.0371 | **0.5453** | 0.1001 | -0.0434 | -0.0330 | **0.1836** | 0.1274 | 0.0540 | -0.0517 | -0.0547 | -0.0173 | 0.0526 | -0.0123 | -0.0570 | -0.0406 | 0.0000 |

Statistically significant values are in bold.
