# Supplementary material for: Incongruent Nuclear and Mitochondrial Genetic Structure of New World Screwworm Fly Populations Due to Positive Selection of Mutations Associated with Dimethyl- and Diethyl-Organophosphates Resistance
Source: PLoS One. 2015 Jun 1;10(6):e0128441. doi: 10.1371/journal.pone.0128441 (PMC4451984; doi:10.1371/journal.pone.0128441)
Supplement: S2 Table — Statistically significant values are in bold. (DOCX) [file pone.0128441.s003.docx]

**S2 Table. Pairwise F_ST_ estimates from mitochondrial data.**

|  | **BTO** | **BGN** | **BGO** | **BCA** | **BCR** | **BAQ** | **BSS** | **BES** | **BCI** | **PYB** | **BFV** | **BSA** | **UST** | **UPM** | **UDA** | **BPM** | **UBM** | **UCC** | **UCO** | **UJS** | **APL** |
| --- | --- | --- | --- | --- | --- | --- | --- | --- | --- | --- | --- | --- | --- | --- | --- | --- | --- | --- | --- | --- | --- |
| **BTO** | 0.0000 |  |  |  |  |  |  |  |  |  |  |  |  |  |  |  |  |  |  |  |  |
| **BGN** | -0.0567 | 0.0000 |  |  |  |  |  |  |  |  |  |  |  |  |  |  |  |  |  |  |  |
| **BGO** | 0.0454 | 0.0529 | 0.0000 |  |  |  |  |  |  |  |  |  |  |  |  |  |  |  |  |  |  |
| **BCA** | 0.1252 | **0.1664** | 0.0460 | 0.0000 |  |  |  |  |  |  |  |  |  |  |  |  |  |  |  |  |  |
| **BCR** | **0.3656** | **0.4038** | **0.3159** | **0.1321** | 0.0000 |  |  |  |  |  |  |  |  |  |  |  |  |  |  |  |  |
| **BAQ** | 0.1317 | **0.1854** | 0.0741 | -0.0438 | 0.0911 | 0.0000 |  |  |  |  |  |  |  |  |  |  |  |  |  |  |  |
| **BSS** | 0.1241 | **0.1374** | -0.0228 | 0.0789 | **0.3250** | 0.0952 | 0.0000 |  |  |  |  |  |  |  |  |  |  |  |  |  |  |
| **BES** | 0.0124 | 0.0038 | -0.0465 | 0.0789 | **0.3662** | 0.1138 | -0.0120 | 0.0000 |  |  |  |  |  |  |  |  |  |  |  |  |  |
| **BCI** | 0.0775 | 0.1021 | -0.0076 | -0.0573 | **0.1782** | -0.0065 | 0.0025 | 0.0053 | 0.0000 |  |  |  |  |  |  |  |  |  |  |  |  |
| **PYB** | -0.0390 | -0.0076 | 0.0307 | 0.0453 | **0.3032** | 0.0659 | 0.1125 | -0.0150 | 0.0225 | 0.0000 |  |  |  |  |  |  |  |  |  |  |  |
| **BFV** | -0.0138 | 0.0275 | 0.0156 | 0.0738 | **0.3352** | 0.0774 | 0.0693 | -0.0082 | 0.0440 | -0.0188 | 0.0000 |  |  |  |  |  |  |  |  |  |  |
| **BSA** | **0.1040** | **0.1109** | **0.1348** | 0.0879 | **0.3321** | **0.1325** | **0.2109** | **0.1160** | 0.0790 | 0.0245 | **0.1088** | 0.0000 |  |  |  |  |  |  |  |  |  |
| **UST** | 0.0480 | 0.0737 | 0.0390 | -0.0266 | **0.2066** | 0.0134 | 0.0967 | 0.0233 | -0.0295 | -0.0449 | 0.0392 | 0.0028 | 0.0000 |  |  |  |  |  |  |  |  |
| **UPM** | 0.0005 | 0.0328 | -0.0497 | -0.0129 | **0.2374** | 0.0000 | -0.0116 | -0.0425 | -0.0406 | -0.0329 | -0.0462 | 0.0918 | -0.0172 | 0.0000 |  |  |  |  |  |  |  |
| **UDA** | **0.2118** | **0.2478** | **0.1623** | -0.0329 | 0.0906 | -0.0124 | 0.1913 | **0.1941** | 0.0212 | 0.1264 | **0.1763** | **0.1399** | 0.0312 | 0.0925 | 0.0000 |  |  |  |  |  |  |
| **BPM** | 0.0264 | 0.0150 | -0.0051 | **0.1772** | **0.4211** | **0.1871** | 0.0246 | -0.0333 | 0.0929 | 0.0607 | 0.0369 | **0.2008** | **0.1287** | 0.0156 | **0.2813** | 0.0000 |  |  |  |  |  |
| **UBM** | -0.0351 | -0.0300 | 0.0706 | **0.1448** | **0.3859** | **0.1642** | **0.1709** | 0.0191 | **0.1074** | -0.0430 | 0.0261 | **0.0930** | 0.0431 | 0.0336 | **0.2233** | 0.0668 | 0.0000 |  |  |  |  |
| **UCC** | -0.0411 | -0.0399 | 0.0160 | 0.1025 | **0.3432** | **0.1235** | 0.1124 | -0.0159 | 0.0650 | -0.0402 | -0.0108 | 0.0751 | 0.0240 | -0.0107 | **0.1885** | 0.0287 | -0.0367 | 0.0000 |  |  |  |
| **UCO** | 0.0689 | 0.0487 | 0.0119 | 0.0960 | **0.3412** | **0.1418** | 0.0370 | -0.0158 | 0.0225 | 0.0747 | 0.0778 | **0.1309** | 0.0710 | 0.0362 | **0.1746** | 0.0376 | **0.1025** | 0.0620 | 0.0000 |  |  |
| **UJS** | 0.0044 | 0.0407 | 0.1055 | **0.1558** | **0.3828** | **0.1551** | **0.2073** | 0.0968 | **0.1462** | 0.0084 | 0.0147 | **0.1412** | 0.0903 | 0.0479 | **0.2337** | **0.1251** | 0.0195 | 0.0061 | **0.1738** | 0.0000 |  |
| **APL** | 0.0364 | 0.0211 | **0.2786** | **0.4107** | **0.6883** | **0.3945** | **0.3987** | **0.2032** | **0.3389** | 0.0930 | 0.1732 | **0.2643** | **0.2484** | 0.2323 | **0.5064** | **0.1871** | 0.0178 | 0.0437 | **0.2576** | 0.0578 | 0.00000 |

Statistically significant values are in bold.
