# Supplementary material for: Incongruent Nuclear and Mitochondrial Genetic Structure of New World Screwworm Fly Populations Due to Positive Selection of Mutations Associated with Dimethyl- and Diethyl-Organophosphates Resistance
Source: PLoS One. 2015 Jun 1;10(6):e0128441. doi: 10.1371/journal.pone.0128441 (PMC4451984; doi:10.1371/journal.pone.0128441)
Supplement: S3 Table — Additionally, mitochondrial haplotypes related to each carboxylesterase E3 (ChαE7) haplotype are shown. E3 hap: carboxylesterase E3 haplotypes; Mit hap: mitochondrial haplotypes. (DOCX) [file pone.0128441.s004.docx]

**S3 Table. Amino acids residues observed in positions 137 and 251 for 49 haplotypes of carboxylesterase E3 (*ChαE7*) obtained from field samples.** Additionally, mitochondrial haplotypes related to each carboxylesterase E3 (***ChαE7***) haplotype are shown. E3 hap: esterase E3 haplotypes; Mit hap: mitochondrial haplotypes.

| **E3 hap** | **Amino acid** | | **Mit hap** | **E3 hap** | **Amino acid** | | **Mit hap** |
| --- | --- | --- | --- | --- | --- | --- | --- |
|  | **137** | **251** |  |  | **137** | **251** |  |
| **1** | Asp | Trp | 5, 6, 14, 15, 21, 25, 27, 31, 42, 43, 50, 59, 65, 68, 71, 72, 78, 81, 83, 85, 86, 99, 100, 111, 114, 115, 119, 122, 128, 137, 140, 143, 146, 154, 162, 167, 171, 176, 178, 179, 183, 185, 186, 187, 188, 289, 290, 291, 293, 298, 299, 301 | **18** | Gly | Trp | 140 |
|  |  |  |  | **19** | Gly | Trp | 128, 301 |
|  |  |  |  | **20** | Gly | Trp | 105 |
|  |  |  |  | **21** | Gly | Trp | 140 |
|  |  |  |  | **22** | Gly | Trp | 140 |
|  |  |  |  | **23** | Asp | Trp | 140 |
| **2** | Gly | Ser | 1, 9, 15, 16, 19, 21, 27, 41, 43, 49, 51, 52, 53, 59, 63, 64, 66, 68, 75, 77, 78, 80, 82, 83, 96, 97, 99, 103, 108, 111, 112, 120, 125, 134, 139, 140, 143, 145, 147, 150, 152, 155, 159, 165, 166, 167, 169, 170, 176, 177, 178, 179, 185, 187, 190, 191, 192, 193, 283, 285, 286, 287, 288, 290, 292, 295, 296, 297 | **24** | Gly | Trp | 152 |
|  |  |  |  | **25** | Asp | Trp | 140 |
|  |  |  |  | **26** | Asp | Trp | 143 |
|  |  |  |  | **27** | Asp | Trp | 143 |
|  |  |  |  | **28** | Asp | Trp | 50 |
|  |  |  |  | **29** | Gly | Trp | 122 |
|  |  |  |  | **30** | Gly | Trp | 140 |
|  |  |  |  | **31** | Asp | Trp | 170 |
| **3** | Asp | Trp | 1, 15, 21, 27, 43, 53, 59, 64, 68, 75, 78, 83, 99, 125, 140, 143, 152, 159, 166, 167, 169, 170,178, 179, 187, 285, 287, 290, 295, 296 | **32** | Asp | Trp | 9 |
|  |  |  |  | **33** | Gly | Trp | 78 |
|  |  |  |  | **34** | Gly | Trp | 121 |
|  |  |  |  | **35** | Gly | Ser | 121 |
| **4** | Gly | Trp | 78, 140, 188, 284 | **36** | Gly | Trp | 78 |
| **5** | Gly | Leu | 78, 179, 186, 187, 188 | **37** | Gly | Trp | 140 |
| **6** | Asp | Trp | 50, 52, 140 | **38** | Asp | Trp | 285 |
| **7** | Gly | Trp | 141, 142 | **39** | Asp | Trp | 185 |
| **8** | Asp | Trp | 50, 113, 146 | **40** | GGC | Trp | 300 |
| **9** | Gly | Trp | 71 | **41** | GGC | Trp | 300 |
| **10** | Gly | Trp | 71, 72 | **42** | Asp | Trp | 136 |
| **11** | Gly | Ser | 23 | **43** | Asp | Trp | 113 |
| **12** | Asp | Trp | 14 | **44** | Gly | Trp | 140 |
| **13** | Gly | Ser | 118 | **45** | Gly | Trp | 78 |
| **14** | Gly | Trp | 78 | **46** | Gly | Trp | 296 |
| **15** | Gly | Ser | 140 | **47** | Asp | Trp | 187 |
| **16** | Gly | Trp | 189 | **48** | Gly | Trp | 81 |
| **17** | Gly | Trp | 141 | **49** | Asp | Trp | 140 |

E3 hap: carboxylesterase E3 haplotypes; Mit hap: mitochondrial haplotypes.
